# Supplementary material for: Oral Anticoagulant Therapy—When Art Meets Science
Source: J Clin Med. 2019 Oct 21;8(10):1747. doi: 10.3390/jcm8101747 (PMC6832236; doi:10.3390/jcm8101747)
Supplement: Supplementary File 1 [file jcm-08-01747-s001.pdf]

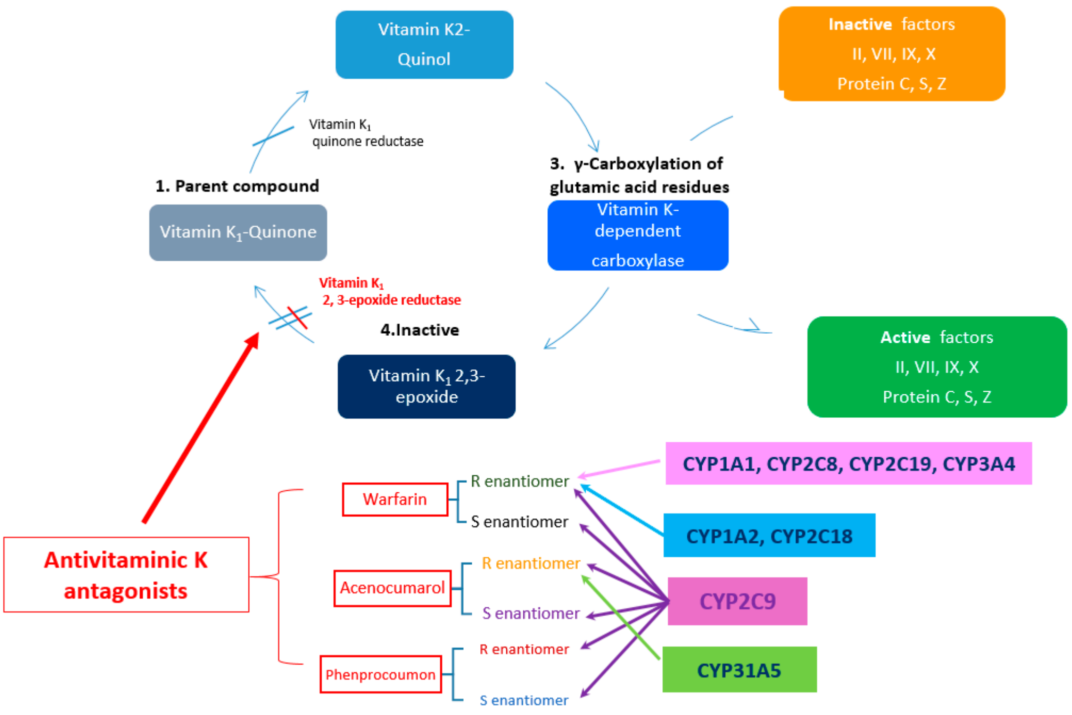

Figure S1. Mechanism of action of antivitaminic K antagonists. CYP1A1 Cytochrome P450 family 1, subfamily A, polypeptide 1; CYP1A2 Cytochrome P450 family 1, subfamily A, polypeptide 2; CYP2C18 Cytochrome P450 family 2, subfamily C, polypeptide 18; CYP2C19 Cytochrome P450 family 2, subfamily C, polypeptide19; CYP3A4 Cytochrome P450 family 3, subfamily A, polypeptide 4; CYP2C9 Cytochrome P450 family 2, subfamily C, polypeptide 9; CYP2C3A5 Cytochrome P450 family 3, subfamily A, polypeptide 5. Adapated after [19] [20]

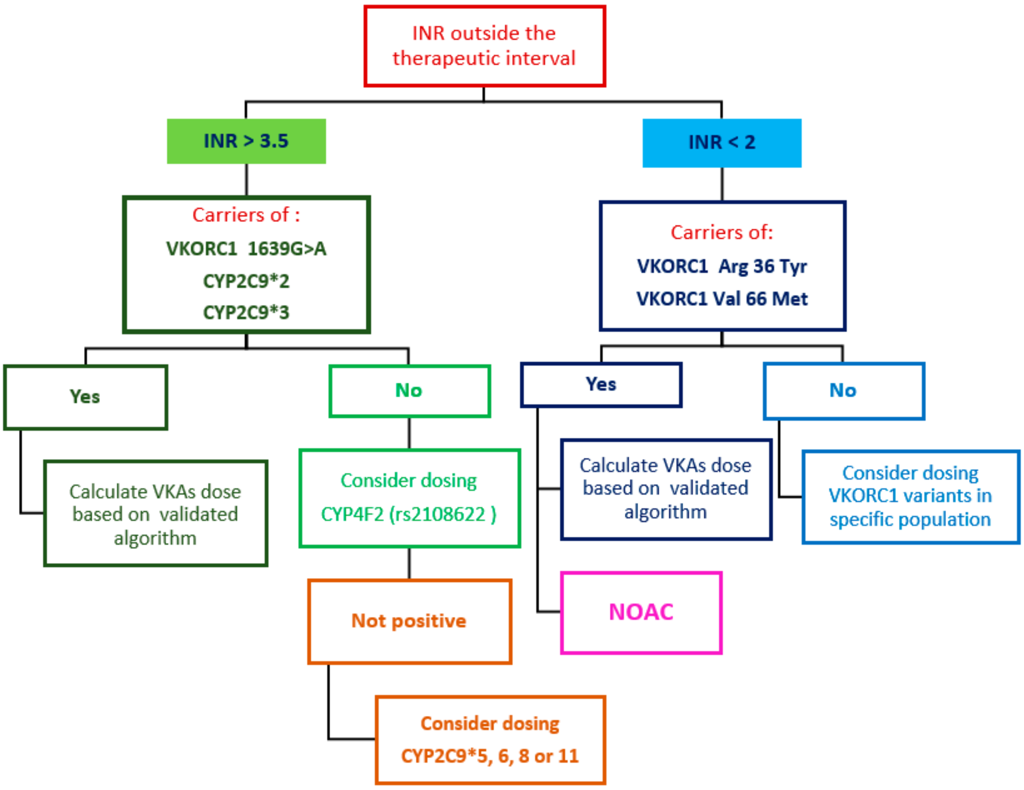

Figure S2. Proposed algorithm for managing INR variability after excluding enviromental factors. Adapted after [71]
